# Three-dimensional low shear culture of *Mycobacterium bovis* BCG induces biofilm formation and antimicrobial drug tolerance

**DOI:** 10.1038/s41522-021-00186-8

**Published:** 2021-02-01

**Authors:** Daire Cantillon, Justyna Wroblewska, Ian Cooper, Melanie J. Newport, Simon J. Waddell

**Affiliations:** 1 Global Health and Infection, Brighton and Sussex Medical School, University of Sussex, Brighton, BN1 9PX UK; 2 School of Pharmacy & Biomolecular Sciences, University of Brighton, Brighton, BN2 4GJ UK

**Keywords:** Biological techniques, Biofilms, Antimicrobials, Pathogens, Bacteriology

## Abstract

Mycobacteria naturally grow as corded biofilms in liquid media without detergent. Such detergent-free biofilm phenotypes may reflect the growth pattern of bacilli in tuberculous lung lesions. New strategies are required to treat tuberculosis, which is responsible for more deaths each year than any other bacterial disease. The lengthy 6-month regimen for drug-sensitive tuberculosis is necessary to remove antimicrobial drug tolerant populations of bacilli that persist through drug therapy. The role of biofilm-like growth in the generation of these sub-populations remains poorly understood despite the hypothesised clinical significance and mounting evidence of biofilms in pathogenesis. We adapt a three-dimensional Rotary Cell Culture System to model *M. bovis* BCG biofilm growth in low-shear detergent-free liquid suspension. Importantly, biofilms form without attachment to artificial surfaces and without severe nutrient starvation or environmental stress. Biofilm-derived planktonic bacilli are tolerant to isoniazid and streptomycin, but not rifampicin. This phenotypic drug tolerance is lost after passage in drug-free media. Transcriptional profiling reveals induction of cell surface regulators, *sigE* and *BCG_0559c* alongside the ESX-5 secretion apparatus in these low-shear liquid-suspension biofilms. This study engineers and characterises mycobacteria grown as a suspended biofilm, illuminating new drug discovery pathways for this deadly disease.

## Introduction

*Mycobacterium tuberculosis* (*M. tb*), the causative agent of tuberculosis (TB), was responsible for 1.5 million deaths in 2018^1^. The 6-month treatment for drug-sensitive TB combines four antimicrobial drugs: isoniazid, rifampicin, pyrazinamide and ethambutol. However, one third of all antimicrobial drug resistant (AMR) infections globally are caused by *M. tb*^2^. Drug-resistant TB often requires longer treatment with less effective and more toxic drug combinations, such as the aminoglycoside streptomycin^3^. The necessity of lengthy drug therapy is attributed to the presence of phenotypically antimicrobial drug tolerant *M. tb* sub-populations that arise due to *M. tb* metabolic and respiratory adaptations to the diverse intracellular and extracellular microenvironments encountered during infection^4,5^. Phenotypic drug tolerance is a temporary, reversible state in bacteria often linked to reduction in cell division rate, where antimicrobial drugs are ineffective despite bacilli being genetically susceptible^6^. Drug tolerant bacilli have been identified in ex vivo leporine lung lesion caseum and in sputa from TB patients^7–10^. A number of in vitro models have been developed to mimic clinically relevant microenvironments to determine the impact of hypoxia or nutrient starvation on *M. tb* physiological state and drug efficacy^11–13^. However, these models alone do not capture the complexity of *M. tb* growth in lung lesions, where anti-TB drugs are directed.

Mycobacteria have long been recognised to grow in macroscopic aggregates in vivo and in vitro, as long chains of cells, known as cording. Georges Canetti described *M. tb* growth as “exuberant” multicellular structures reminiscent of biofilms in histological examination of human TB lung lesions^14^. *M. tb* has also been observed growing as clusters or microcolonies in the acellular rim of granulomas in the guinea pig model of infection, even after treatment with anti-tuberculous drugs^15^. Such aggregations of mycobacteria likely act as an axenic biofilm, with local microenvironments created by nutrient or oxygen gradients driving bacterial phenotypic diversity and generating potentially antimicrobial drug tolerant populations, recalcitrant to drug therapy. This corded biofilm-like phenotype is overlooked by most in vitro mycobacterial research, where detergent is added to liquid media to enhance bacterial enumeration and increase experimental reproducibility.

Since Ojha et al.^16^ demonstrated that *M. tb* biofilms formed at the liquid-air interface harbour antimicrobial drug tolerant bacilli, research into the role of biofilms in TB pathogenesis has expanded. Mycobacterial biofilm models, often based on the formation of a pellicle at the liquid–air interface, have demonstrated that mycobacteria in biofilm-like structures are less sensitive to antimicrobial drug exposure, show biofilm architecture and an extracellular matrix readily identifiable by microscopy, and express distinct transcriptional profiles when compared to planktonic growth^16–19^. These systems often rely on attachment to artificial surfaces or exposure to stringent environmental conditions to create biofilm-like structures. The use of 3D cell culture systems are increasingly applied across a range of disciplines, as they allow in vivo environments to be more closely replicated in vitro. This enhances the formation of complex cellular structures and microarchitecture, of importance when considering biofilm formation. *M. tb* in an electrospray 3D granuloma model, generated using primary human cells and a collagenous extracellular matrix, exhibited drug sensitivities similar to in vivo drug efficacies, including sensitivity to pyrazinamide that is not observed in standard in vitro culture^20^. Non scaffold-based 3D culture offers increased opportunities for complex structures to emerge in liquid suspension and may be particularly relevant to the study of respiratory pathogens. *Pseudomonas aeruginosa* in ex vivo lung tissues from people with cystic fibrosis formed biofilms that were not surface-attached to host epithelial tissue but free-floating aggregates of cells in the mucus of the bronchial lumen^21^. Non-attached *P. aeruginosa* in vitro biofilms have similar characteristics to in vivo biofilms, including adaptations that result in antimicrobial drug tolerance^22^.

The Rotary Cell Culture System (RCCS) was originally developed by the National Aeronautics and Space Administration (NASA) as a rotating vessel bioreactor to investigate the impact of microgravity on cellular processes^23^. The rotating low shear environment enables growing cell clusters to form with enhanced multicellular structural complexity without surface attachment, alongside high rates of nutrient, waste and gaseous exchange superior to 2D models^24,25^. This ensures that the biological adaptations to biofilm-like growth are in response to nutrient, waste or gas gradients in three dimensions via apical, lateral and basal surfaces of the growing aggregation of cells^26^. A key feature of the RCCS culture system is that 3D cellular structures are allowed to develop without any destructive mechanical shear forces that would be introduced through media changes or aeration in standard culture models^27^. The system has been used to generate complex, hierarchical cellular structures for eukaryotic tissues and for host–pathogen interaction studies^28^.

Here, we developed a three-dimensional liquid suspension cell culture system to model mycobacterial corded biofilm-like growth. *M. bovis* BCG biofilm-derived bacilli were tolerant to key anti-tuberculosis drugs and supernatant from biofilms induced a greater pro-inflammatory macrophage response. Transcriptomics highlighted cell surface regulatory elements and ESX-5 type VII secretion components as likely mediating adaptations to a biofilm substructure. These findings suggest that detergent-free corded biofilm-like growth may facilitate the development of drug tolerant phenotypes observed in vivo.

## Results

### Three-dimensional low shear culture generates a mycobacterial biofilm phenotype

**Fig. 1:**
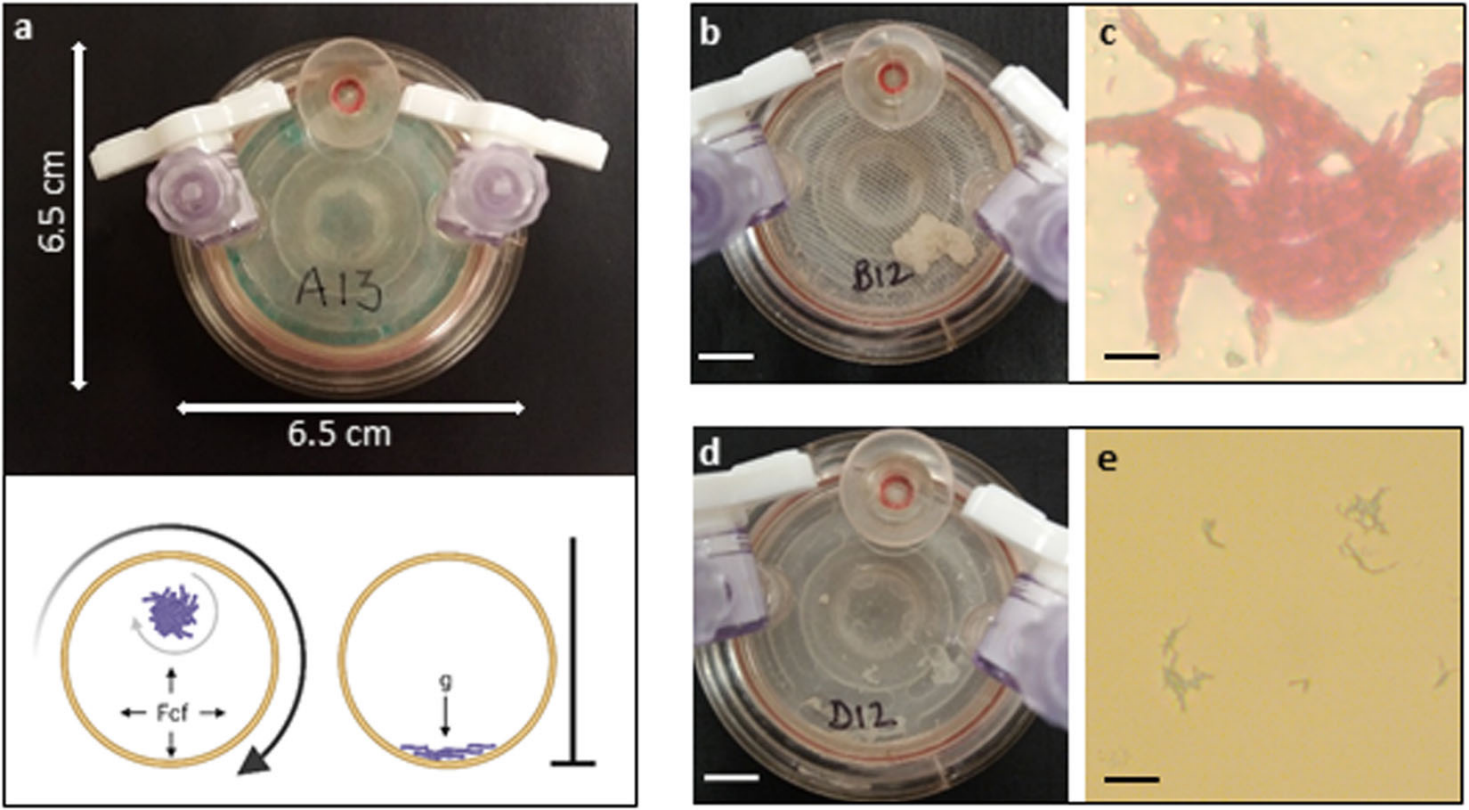
Three-dimensional low shear Rotary Cell Culture System (RCCS) generates a mycobacterial biofilm phenotype. **a** The RCCS disposable vessel with entry ports to the front and gas permeable membrane and rotation fixing to rear. Centrifugal force (Fcf) is controlled through rotation speed enabling biomass to grow without encountering the vessel walls. Gravity (g) in non-rotating controls collects biomass to the bottom of the vessel. **b** *M. bovis* BCG RCCS-biofilm formed after 21 days. **c** Heavily corded Kinyoun-stained RCCS-biofilm after 21 days. **d** Biomass in non-rotating control at the bottom of the vessel after 21 days (vessel agitated for imaging). **e** Kinyoun-stained non-rotating control with limited cording after 21 days. White scale bar is 1 cm, black scale bar is 20 µM.

**Fig. 2:**
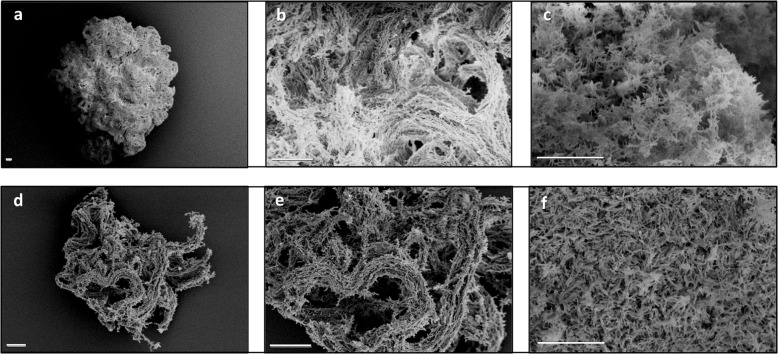
Biofilm macrostructure reveals densely packed bacilli looped into corded structures. Scanning electron microscopy of **a**–**c** RCCS-biofilm and **d**–**f**. non-rotating *M. bovis* BCG control. RCCS-biofilm shows more compact growth with enriched topology and higher deposition of extracellular matrix. Scale bar is 20 µM in all images.

To understand the physiology of *M. tb* bacilli that grow in biofilm-like structures that might mimic extracellular growth in pulmonary lung lesions, we developed a three-dimensional suspended liquid culture biofilm model. The Rotary Cell Culture System (RCCS), first developed by NASA to assay cellular adaptations to microgravity, was optimised for *Mycobacterium bovis* BCG. In this low shear rotary culture system, clusters of bacilli grow together suspended in liquid detergent-free Sauton media. As the aggregation of mycobacteria falls through the vessel, centrifugal and Coriolis effects from rotation counter gravity maintaining the three-dimensional biofilm permanently in suspension (Fig. 1a). Unlike other biofilm models, this system enabled the natural cord-forming mycobacterial phenotype to develop without attachment to an artificial solid medium, nor reliance on an air/liquid interface for pellicle formation^16,18,19,29^. Mycobacterial biofilms were macroscopically visible in rotating RCCS vessels, as was the accumulation of biomass in the bottom of non-rotating vessels by day 21 (Fig. 1b/d). As expected, microscopy revealed extensive cording of acid-fast bacilli in RCCS-biofilm cultures in comparison to non-rotating (control) culture (Fig. 1c/e). Scanning electron microscopy showed a compact and complex biofilm macrostructure, with densely packed bacilli looped into corded structures coated with extracellular material (Fig. 2). RCCS-biofilm growth resulted in a more compact matrix of bacilli with a greater extent of extracellular material. This biofilm phenotype was generated without excessive aging or nutrient starvation. The RCCS vessels are designed to allow passive diffusion of gases through a posterior silicone membrane, permitting mycobacterial colonies to grow as cords suspended in liquid media. The model therefore allowed the direct impact of enhanced biofilm architecture on the formation of antimicrobial drug tolerant phenotypes to be investigated.

### Biofilm-derived bacilli are tolerant to the antimicrobial drugs isoniazid and streptomycin

**Fig. 3:**
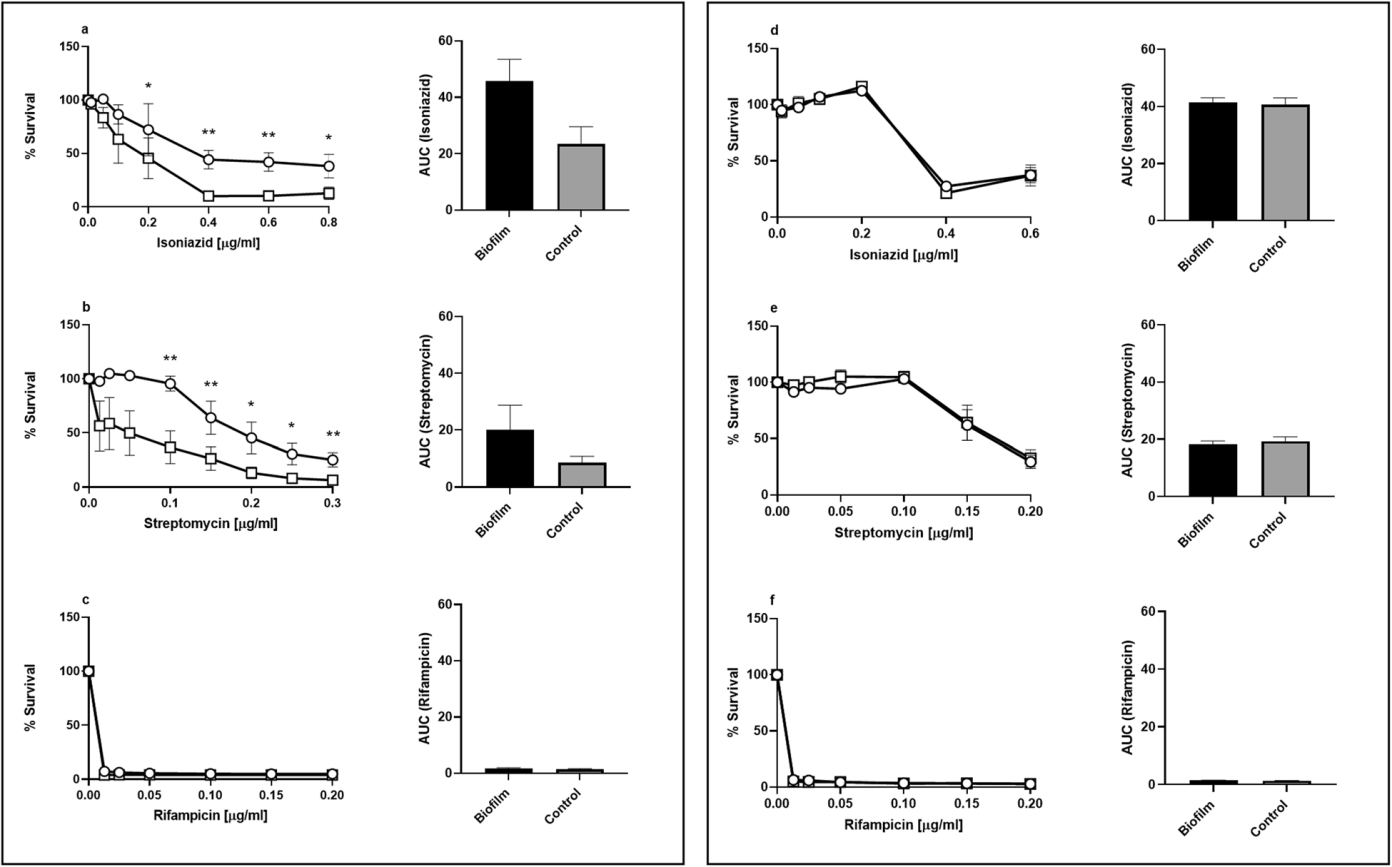
Biofilm-derived bacilli are tolerant to isoniazid and streptomycin. **a**–**c** RCCS-biofilm (circle) or non-rotating control (square) bacilli were homogenised to single cell suspensions and exposed to isoniazid, streptomycin or rifampicin. Biofilm-derived bacilli showed enhanced survival to isoniazid and streptomycin, but not rifampicin. **d**–**f** This drug tolerant phenotype was lost after passage in drug-free media. Area under the curve (AUC) plots illustrating the difference in drug efficacy over multiple drug concentrations are detailed alongside each plot. Percentage survival calculated relative to untreated bacilli. Error bars are ±standard error of the mean from two biological replicates each with duplicate technical replicates. *p value ≤ 0.05; **p value ≤ 0.01.

The clinically relevant antimicrobial drugs, isoniazid, rifampicin and streptomycin (targeting cell wall biosynthesis, transcription and translation, respectively) were used as tools to characterise the metabolic adaptations caused by biofilm formation. RCCS-biofilm and non-rotating control bacilli were first homogenised to single-cell suspensions before assessing antimicrobial drug efficacy, to negate the impact of physical barriers created by biofilm growth. Importantly, we show that planktonic suspensions of bacilli derived from biofilms exhibited drug tolerance to isoniazid and streptomycin, but not rifampicin, in comparison to non-rotating control cultures (Fig. 3a–c). Reduction in isoniazid and streptomycin antimicrobial activity was observed at multiple drug concentrations. We hypothesise that the compact cording seen in RCCS-biofilm cultures (Fig. 2) generates greater populations of drug-tolerant bacilli, which remain phenotypically drug-tolerant even as single cells in suspension. To demonstrate that this loss of cidal drug action was phenotypically mediated rather than the emergence of genetically encoded drug resistance, RCCS-biofilm and non-rotating control cultures were passaged three times in drug-free media (with detergent) before assaying drug activity as before. All bacilli were equally sensitive to the three antimicrobial drugs (Fig. 3d–f), establishing that loss of drug activity against biofilm-derived bacilli was due to phenotypic adaptations that induced drug tolerance. Biofilm formation affords bacteria protection from environmental insults including antimicrobial drug exposure, and previous mycobacterial biofilm models have demonstrated drug tolerance^16,19^. These models treated biofilms directly with antimicrobial drugs without homogenisation, so the impact of biofilm formation on mycobacterial phenotype, as opposed to drug penetration into the biofilm, was not directly assessed as in this study.

Biofilm-derived cells were tolerant to isoniazid and streptomycin but not rifampicin. Isoniazid, a first line antitubercular drug, is most effective against replicating bacilli, where the FasII cycle target is essential for cell wall biosynthesis^30^. Drug tolerance is observed in non-replicating or slowly replicating populations, where the target likely becomes non-essential to mycobacterial viability^31^. Reduced isoniazid efficacy in this model suggests that RCCS-biofilm growth generates greater proportions of non- or slowly-replicating populations of bacilli, or creates populations of bacilli with modified cell wall constituents, that are tolerant to isoniazid even after the substructure of the biofilm is destroyed. Streptomycin has some bactericidal activity against non-dividing stationary phase bacteria^32^, however we observed a reduction in drug efficacy in biofilm-derived relative to non-rotating control bacilli. The presence of a biofilm-associated extracellular matrix has been shown to reduce aminoglycoside efficacy in *P. aeruginosa*^33^, and streptomycin exerts poor activity against intracellular mycobacteria^5^. Further experiments will be required to determine if streptomycin tolerance in this model is due to decreased penetration of drug or reduced importance of the target pathway. Rifampicin, a first line TB drug, has been shown to be highly effective against *M. tb* biofilms, reducing viability >1000 fold, but failing to eradicate a subset of bacilli that persisted through drug exposure^16^. Rifampicin tolerance was not observed in our RCCS-biofilm model, showing that a large proportion of bacilli found in the biofilm structure were transcriptionally active. This underscores the novelty of this system, enabling corded mycobacterial growth without extensive nutrient depletion steps or exogenous environmental stress. The drug-tolerant phenotype demonstrated here, derived from enhanced mycobacterial aggregation, may mimic *M. tb* biofilm formation that has been observed in lung lesions^14,15,34^, in potentially nutrient replete surroundings where bacilli have adapted to replicate successfully.

### The biofilm transcriptome reveals key regulatory adaptations to the cell surface

**Fig. 4:**
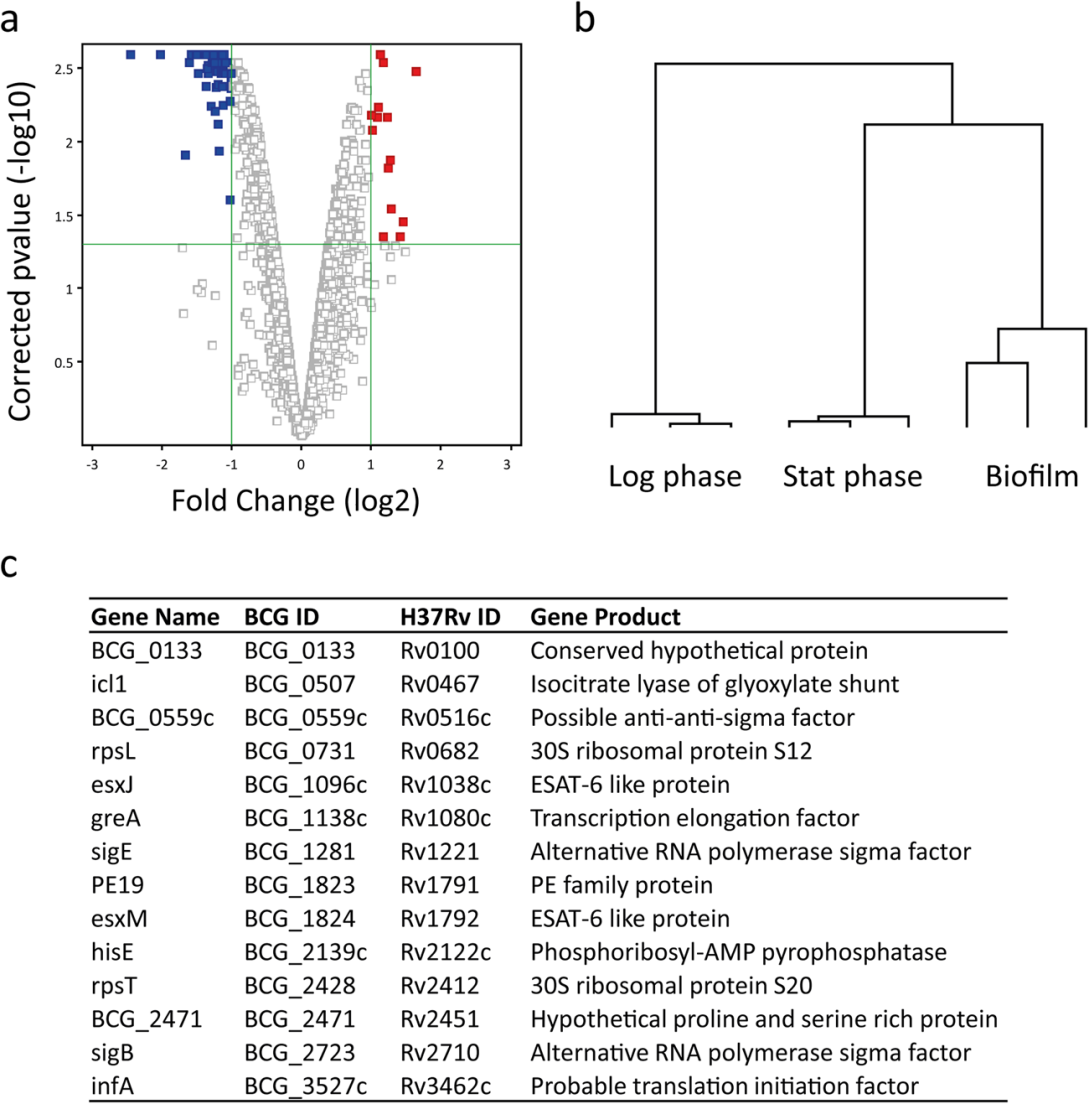
Mycobacterial transcriptional response to growth as a corded biofilm. **a** Volcano plot showing transcriptional adaptations to biofilm growth comparing RCCS-biofilm to stationary phase bacilli (red, induced in biofilm; blue, repressed). **b** Dendrogram of three biological replicates of RCCS-biofilm alongside log phase and stationary phase controls. Hierarchical clustering of mean-centred significantly differentially expressed genes to highlight differences between RNA signatures. **c** Genes significantly induced by biofilm growth, detailing *M. bovis* BCG and *M. tb* identifiers. Ordered by BCG ID.

To understand the impact on gene expression of growth in an enhanced corded biofilm, the transcriptome of RCCS-biofilm bacilli was contrasted to standardised log (day 5) and stationary phase (day 21) bacilli grown in Sauton media without detergent. The RNA profiles of biofilm-derived bacilli clustered most closely to stationary phase *M. bovis* BCG signatures (Fig. 4a/b). In addition, the average Pearson correlation, measuring the similarity between conditions, of RCCS-biofilm was most similar to stationary phase (0.89, in comparison with 0.86 to log phase). Therefore, to define adaptations driven specifically by biofilm-like cellular aggregation rather than growth rate, we identified genes differentially expressed in RCCS-biofilm relative to stationary phase bacilli; 14 genes were significantly induced greater than 2-fold in biofilms (Fig. 4a/c). The transcriptional signature of biofilm formation was dominated by genes involved in the regulation of transcription and translation, evidenced by significant enrichment of the gene annotation functional category (II.A) Synthesis and Modification of Macromolecules^35^, and GO term (GO:0008135) Translation Factor Activity, Nucleic Acid Binding (hypergeometric probabilities of 2.71 × 10^−5^ and 1.54 × 10^−5^ respectively). The alternative sigma factors *sigB* and *sigE*, that control mycobacterial responses to environmental cues such as nutrient starvation and cell surface stress, were induced. Interestingly, both *sigB* and *sigE* null mutants are more sensitive to isoniazid and streptomycin but not rifampicin^36^, mirroring our biofilm drug tolerance phenotype and implicating these regulatory factors as key mediators of the adaptations to biofilm growth. *BCG_0559c* (*Rv0516c*), a putative anti-anti-sigma factor, was also upregulated in RCCS-biofilm relative to stationary phase. This regulatory protein likely mediates responses to osmotic stress, with disruption leading to reduced thickness of the peptidoglycan layer in the *M. tb* cell wall^37^. We hypothesise that induction of this putative osmoregulator highlights changes to the microenvironments surrounding bacilli as they grow together in a biofilm-like structure. Of note, these regulators (*sigB*, *sigE* and *Rv0516c*) were also induced in the formation of *M. tb* biofilms generated by exposure to dithiothreitol (DTT)^19^. Also induced in RCCS-biofilm was *greA*, a transcription elongation factor that enables RNA polymerase to overcome blocks caused by RNA-DNA hybrids. Knockdown of *greA* abrogated pellicle formation and reduced biofilm-like growth in *M. smegmatis*^38^. Induction of the translation initiation factor, *infA*, and S12 and S20 30S ribosomal proteins, encoded by *rpsL* (targeted by streptomycin) and *rpsT* respectively, that form part of the translation machinery suggest that bacilli were metabolically active in the enhanced RCCS-biofilm macrostructure.

Genes of the mycobacterial ESX-5 type VII secretion system (*esxJ*, *esxM* and upstream *PE19*) were induced in biofilm-derived compared to stationary phase bacilli. The ESX-5 apparatus, found only in slow-growing mycobacterial species, is responsible for the secretion of multiple substrates including PE/PPE proteins. Of note, ESX-5, in *M. marinum*, has been implicated in the activation of the macrophage inflammasome and the release of pro-inflammatory cytokines, supporting a role for this secretion apparatus in mediating host immune responses^39^. The system has also been demonstrated to be involved in adjusting cell wall permeability and in the uptake of hydrophobic carbon sources^40^. Isocitrate lyase, *icl1*, required in the glyoxylate shunt and methylcitrate cycles that are upregulated in macrophages and in sputa, was also induced^41^, suggesting that biofilm growth drives a lipolytic metabolic response comparable to *M. tb* in vivo. In summary, biofilm formation in free-fall suspension caused adaptations to cell surface control systems, exemplified by induction of *sigE* and *BCG_0559c*, in comparison to stationary phase culture in the absence of detergent. Significantly, *sigE* and elements of the ESX-5 secretion system (*esxJ*, *esxM*, *PE19* found here, plus *esxN*, *BCG_1826* and *esxK*) were also found to be induced when *M. bovis* BCG cells first began to aggregate in a Sauton no detergent model of pellicle formation^18^, highlighting these systems as likely mediators of the dynamic mycobacterial phenotypes generated in biofilm-like growth.

Induction of the ESX-5 secretion apparatus in RCCS-biofilm bacilli suggested that growth as a biofilm may result in the differential secretion of mycobacterial effector molecules, which might alter interactions with host immune cells. Therefore, we tested whether filter-sterilised supernatant from RCCS-biofilm or non-rotating control bacilli was cytotoxic to, or induced differential cytokine release in, differentiated human THP-1 macrophages. Neither supernatants (10% v/v) from day 7 nor day 21 biofilm or control bacilli caused detectable macrophage loss (Supplementary Fig. 2a). There was no significant difference in IL-1β secretion. However, TNFα release was significantly greater after exposure to RCCS-biofilm day 21 supernatant compared to non-rotating control *M. bovis* BCG (Supplementary Fig. 2b). TNFα, a key pro-inflammatory cytokine released by alveolar macrophages, is fundamental to orchestrating granuloma formation in the lung in response to *M. tb*, however overproduction may lead to excessive immunopathology^42^. Greater induction of TNFα by RCCS-biofilm supernatant may suggest that mycobacterial corded growth modifies the secretion of effector molecules that could alter the interactions between host and pathogen. Further biochemical characterization of this response will determine whether it is dependent on the ESX-5 type VII secretion system in *M. tb*.

## Discussion

**Fig. 5:**
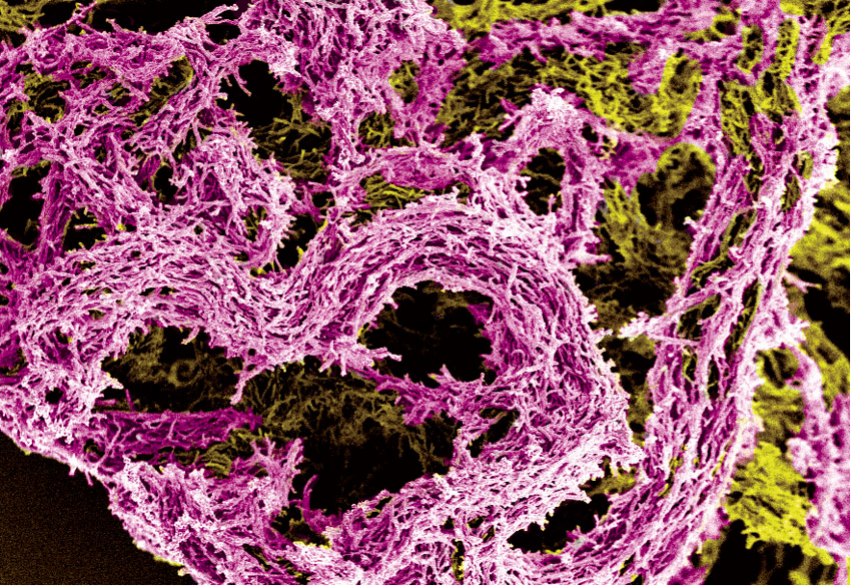
Mycobacterial cording as a biofilm in detergent-free liquid media. A false-colour scanning electron microscopy image of *M. bovis* BCG grown in detergent-free liquid media (magnification ×1000), exemplifying the dense cording phenotype that may better represent *M. tb* lung lesion growth, which is quite different from single cells found in liquid media containing detergent.

An improved understanding of the *M. tb* phenotypes present in vivo will aid rational drug discovery for a disease that continues to newly infect 10 million people each year. Here, we exploited the natural cord-forming ability of *M. bovis* BCG to develop a low shear liquid biofilm model to mimic mycobacterial growth in lung tissue. Importantly, the model allowed bacilli to grow as corded structures in suspension without applying nutrient starvation, aging, or environmental stress. This permitted the phenotype of bacilli grown as a corded biofilm to be defined separate from divergent and overlapping adaptations to dramatic changes in the microenvironment. We hypothesised that biofilm-like growth would generate diverse phenotypes of bacilli as the aggregations of bacteria expanded, analogous to *M. tb* colonies observed in pulmonary lung lesions. Bacilli liberated from biofilms demonstrated phenotypic drug tolerance to the anti-TB drugs isoniazid and streptomycin. Cell surface regulatory factors SigE and BCG_0559c (Rv0516c) and the ESX-5 secretion system were implicated in mediating the response to biofilm growth, and these adaptations resulted in a greater pro-inflammatory response from macrophages exposed to biofilm supernatants. Thus, we conclude that the natural ability to cord is alone sufficient to induce antimicrobial drug tolerance in vitro (Fig. 5). We hypothesise that this phenomenon also generates phenotypic heterogeneity in vivo, leading to populations of bacilli that persist through drug therapy necessitating the current 6-month treatment regimen for TB.

Biofilms are widely implicated in chronic infections in humans. Biofilm formation protects its bacterial inhabitants against environmental insults, antimicrobial agents, and the host immune response, and it is a key pathogenic process for many bacteria. Biofilms are implicated in urinary tract, soft tissue, pulmonary and surgical implant infections^43–45^. Traditional 2D culture poorly predicts clinical activity of new drugs, and the development of 3D culture methods that more accurately reflect the in vivo environment offers new avenues for drug discovery^46^. The addition of detergents to mycobacterial media is common practice to encourage homogenous bacterial culture, and while this facilitates reproducible in vitro research, it does not accurately reflect the growth state of mycobacteria in vivo. The development of new tools to engineer, characterise and screen mycobacterial biofilms in detergent-free systems^18,47^ may lead to the discovery of new transformative drugs targeted where they are required, at drug-tolerant sub-populations of bacilli residing in pulmonary lung lesions.

## Methods

### Bacterial culture

*Mycobacterium bovis* BCG Pasteur was cultured at 37 °C in Middlebrook 7H9 broth (Sigma Aldrich) supplemented with albumin dextrose catalase (ADC) (10% v/v) and Tween 80 (0.05% v/v), or in Sauton media with or without Tween 80 (0.05% v/v). Sauton media was prepared by dissolving 0.5 g potassium phosphate, 0.5 g magnesium sulphate, 4 g l-asparagine, 2 g citric acid, 0.05 g ferric ammonium citrate and 60 ml glycerol in 900 ml of water. The pH was adjusted to 7.2 before autoclaving at 121 °C for 15 min and addition of 100 µL sterile 1% zinc sulphate. Optical density was measured using a spectrophotometer at absorbance 600 nm. Colony forming units (CFU) were determined by serially diluting cultures onto Middlebrook 7H10 agar (Sigma Aldrich) supplemented with 0.5% glycerol and oleic acid albumin dextrose catalase (10% v/v) and incubating at 37 °C for four weeks. Microscopy for acid-fast bacilli was performed by Kinyoun staining (Becton Dickinson GmbH, Oxford, UK) following manufacturer’s instructions. Bacilli were imaged at 40X magnification using an Axiovert 40CFL light microscope (Zeiss, Cambridge, UK). Images were captured using Axiovision SE64 software, version 4.9.1.

### Biofilm model

Mycobacterial biofilms were generated using the Rotary Cell Culture System (RCCS-4DQ) with power supply and tachometer to independently control the rotation of four disposable High Aspect Ratio (HARV) bioreactor vessels (Synthecon, Houston, USA). To prepare the RCCS inoculum, *M. bovis* BCG was passaged from frozen stock into Middlebrook 7H9 ADC with Tween 80, then cultured for 7 days in Sauton media with Tween 80 (Supplementary Fig. 1). The use of defined Sauton media will allow media constituents to be easily manipulated, for example modifying carbon and nitrogen sources. This culture was diluted to an OD 600 nm 0.05–0.06 (10^6^ cells/ml) with Sauton media no Tween 80 before adding to the RCCS vessels that had been equilibrated with sterile PBS overnight. The vessels were incubated with (RCCS) or without (control) rotation at 15 rpm in a 37 °C humidified incubator. The rotation of the vessels was adjusted to counter the sedimentation of growing mycobacterial clusters pulled downwards by gravity, allowing biofilms to form in a low shear environment through continuous suspension. The cultures (RCCS-biofilms and non-rotating controls) were harvested on day 21.

### Scanning electron microscopy

Samples were fixed for scanning electron microscopy (SEM) with 100% glutaraldehyde at 2.5% v/v final volume and incubated at room temperature for 2 h. The samples were dehydrated by critical point drying using a graded series of ethanol. After sputter-coating with gold, samples were imaged using a Leica Leo Stereoscan 420 scanning electron microscope at 5 kV with 10pA probe current x 124 to 5000.

### Determination of antimicrobial drug efficacy

Isoniazid and streptomycin were prepared as 10 mg/ml stock solutions in sterile dimethyl sulfoxide (DMSO). Rifampicin was prepared in 90% w/v methanol. RCCS-derived biofilm and non-rotating control cultures were needle homogenised to planktonic suspensions and adjusted to OD 600 nm 0.005, corresponding to 10^5^ CFU/ml. Microtitre plates containing ranges of antimicrobial drug concentrations in duplicate were inoculated and incubated for seven days at 37 °C. CellTiter-Blue (Promega) was added at a final concentration of 10% v/v and incubated overnight. Fluorescence was measured at excitation 580–640 nm and emission 520 nm using a Synergy HT plate reader. Fluorescence data were corrected for background using media-only controls. Percentage survival was calculated relative to untreated bacilli. To determine between phenotypic drug tolerance or the emergence of genetically-encoded drug resistance, the RCCS-biofilm and non-rotating control inoculums (prepared for the drug efficacy assays) were passaged three times in drug-free 7H9 ADC media (with Tween), before reassessing drug activity as described above.

### RNA extraction and mycobacterial transcriptomics

Mycobacteria were recovered using a guanidine thiocyanate (GTC)/Trizol extraction method as previously described^48^. RNA was extracted by bead-beating with 0.1 mm silica beads for 45 s at speed 6.5 m/s (MP Biomedicals, Santa Ana, USA), followed by chloroform extraction and RNA purification using the mirVana RNA isolation kit (ThermoFisher Scientific). Mycobacterial RNA was treated with DNase I (Primerdesign, Southampton, UK), and RNA yield and quality assessed using the NanoDrop ND-1000 Spectrophotometer (NanoDrop Technologies) and Agilent 2100 Bioanalyzer (Agilent Technologies). RNA samples were directly labelled with Cy3 fluorophore using the Universal Linkage System (ULS, Kreatech Diagnostics), as previously described^49^, and hybridised to high density Agilent tiling arrays with 180,000 60-mer oligonucleotides evenly tiled across the *M. tb* H37Rv genome, designed by the Bacterial Microarray Group at St George’s, University of London (ArrayExpress accession A-BUGS-47). Expression ratios were generated by averaging the antisense probes for each gene in the *M. bovis* BCG Pasteur genome. RNA from three independent biological replicates of RCCS-biofilm were contrasted to log phase (day 5; OD 0.400 ± 0.053, ~7 × 10^6^ CFU/mL) and stationary phase (day 21, OD 0.580 ± 0.040, ~2 × 10^8^ CFU/mL) bacilli, cultured in triplicate in static Sauton media no Tween 80.

### Macrophage culture and cytokine quantification

Human THP-1 monocytes were cultured in RPMI 1640 medium supplemented with 10% v/v heat inactivated foetal calf serum and 2 mM l-glutamine at 37 °C in a 5% CO_2_ humidified incubator. THP-1 cells were differentiated into phagocytic macrophage-like cells by stimulation with 20 ng/ml (final concentration) phorbol 12-myristate 13-acetate overnight. The macrophage monolayer was washed and incubated for a further 48 h before exposure to mycobacterial culture supernatants for 24 h. Supernatants (filter-sterilised after removal from RCCS-biofilm and non-rotating control vessels) or media only controls were diluted to 10%, 25% or 50% v/v with RPMI for viability testing or 10% v/v for cytokine Enzyme-Linked Immunosorbent Assays (ELISA) assays. THP-1 cell viability was assessed using CellTiter-Blue as described above, incubating for 2 h before data acquisition. ELISA assays determined IL-1β and TNFα release after macrophage exposure to mycobacterial culture supernatants or 10 ng/ml lipopolysaccharide (LPS), using matched (capture and detection antibody pair) anti-IL-1β and anti-TNFα human antibodies (R&D Systems). Streptavidin horseradish peroxidase conjugate was used with its chromogenic substrate 3, 3′, 5, 5′-tetramethylbenzidine. Absorbance was measured at 450 nm with a Synergy HT plate reader with wavelength correction of 540 nm; concentrations were determined from IL-1β and TNFα standard curves.

### Statistical methods

Statistically significant differences in drug efficacy between RCCS-biofilm and non-rotating controls were determined using a paired parametric t-test from two biological replicates each with 2–3 technical replicates per antimicrobial drug concentration using GraphPad Prism 8. Area under the curve (AUC) for these plots was determined by multiplying % survival by the drug concentration in µg/ml, baseline *Y* = 0 in GraphPad Prism 8. Pearson correlation was applied to measure the similarity in transcriptional pattern between growth conditions, calculating the average correlation of biological replicates between conditions from 3,752 gene expression ratios. Significantly differentially expressed genes were identified using a moderated *t*-test (*p*-value < 0.05 with Benjamini and Hochberg multiple testing correction) and fold change >2 in GeneSpring 12.6 (Agilent Technologies) comparing RCCS-biofilm bacilli to stationary phase bacilli (Supplementary Table 1). Significant overlaps in transcriptional signatures were identified using hypergeometric probability. Statistical significance in cytokine ELISAs was determined using an unpaired parametric *t*-test, *p* value < 0.01.

### Reporting summary

Further information on research design is available in the Nature Research Reporting Summary linked to this article.

## Supplementary information

Supplementary Information

Reporting Summary

## Data Availability

All data relevant to the article is included in the article and its supplementary information. Fully annotated microarray data have been deposited in ArrayExpress (accession number E-MTAB-9904).
