# Supplementary material for: Three-dimensional low shear culture of Mycobacterium bovis BCG induces biofilm formation and antimicrobial drug tolerance
Source: NPJ Biofilms Microbiomes. 2021 Feb 1;7:12. doi: 10.1038/s41522-021-00186-8 (PMC7851154; doi:10.1038/s41522-021-00186-8)
Supplement: Supplementary file 1 — Supplementary Information [file 41522_2021_186_MOESM1_ESM.pdf]

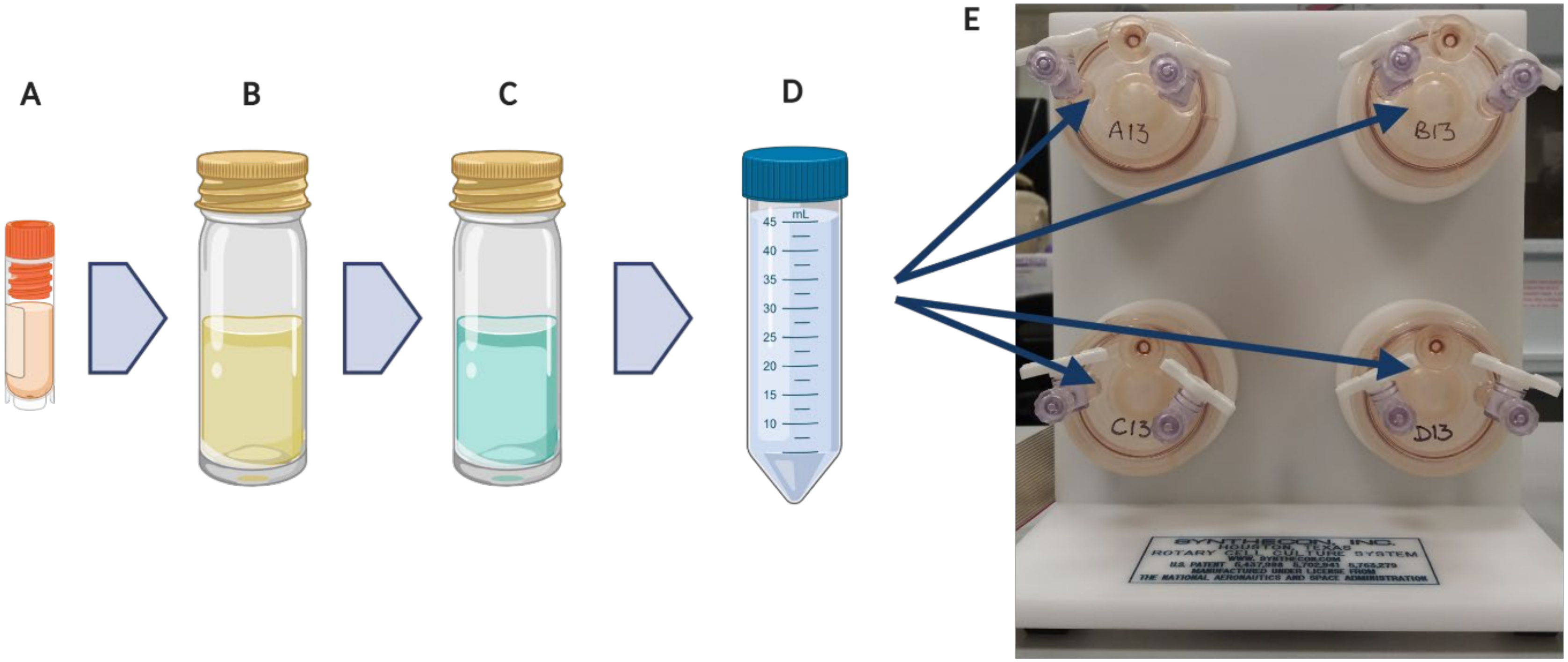

Supplementary Figure 1: Preparation of the *M. bovis* BCG RCCS-biofilm.

A glycerol stock **(A)** of *M. bovis* BCG was passaged in Middlebrook 7H9 ADC with 0.05% Tween 80 **(B)** for seven days. This was then passaged in Sauton media with 0.05% Tween 80 **(C)** for seven days, before diluting to ~1x10^6 CFU/mL **(D)** in Sauton media (no Tween 80), which was used to inoculate the RCCS vessels **(E)**. The top two vessels were rotated to facilitate biofilm formation, while the lower two vessels remained static as non-rotating paired controls. Created with BioRender.

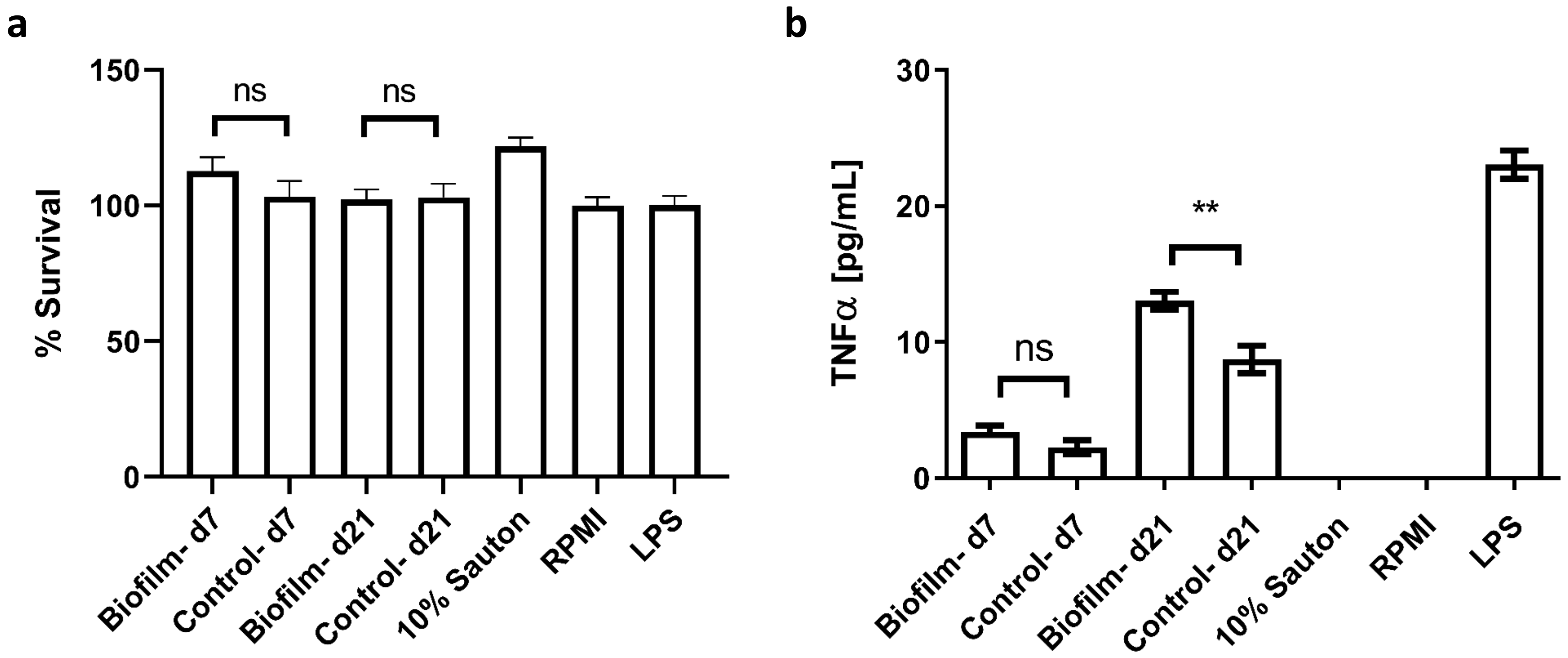

Supplementary Figure 2: Biofilm supernatants are not cytotoxic and induce pro-inflammatory TNFα secretion.

THP-1 macrophage-like cells were exposed to RCCS-biofilm and non-rotating control supernatants harvested at days 7 and 21. **(a)** Biofilm or control supernatants were not toxic to macrophages. **(b)** Day 21 biofilm supernatant significantly induced TNFα secretion compared to controls. Percentage survival calculated relative to untreated bacilli. Data points are from three biological replicates and statistical significance determined by t-test ** = p value ≤0.01. Error bars are ± standard error of the mean.

Supplementary Table 1: Transcriptional response to *M. bovis* BCG corded biofilm growth.

Differentially expressed genes were identified using a modified t-test with Benjamini-Hochberg multiple testing correction (adjusted p value <0.05) with fold change >2 comparing RCCS-biofilm to stationary phase bacilli. Table ordered by BCG identifier with updated *M. tb* H37Rv annotation from Mycobrowser.

BCG ID	BCG Name	H37Rv ID	Rv Name (MB)	Regulation	FC (abs)	p (Corr)	Function (MB)	Product (MB)	Functional Category (MB)	Target_ID
BCG_0133	BCG_0133	Rv0100	Rv0100	up	2.4253385	0.028632747	Function unknown	Conserved hypothetical protein	conserved hypotheticals	Rv0100-R
BCG_0507	icl	Rv0467	icl1	up	2.0022416	0.006544662	Involved in glyoxylate bypass (at the first step), an alternative to the tricarboxylic acid cycle (in bacteria, plants, and fungi) [catalytic activity: isocitrate = succinate + glyoxylate]. Involved in the persistence in the host.	Isocitrate lyase Icl (isocitrase) (isocitratase)	intermediary metabolism and respiration	Rv0467-R
BCG_0559c	BCG_0559c	Rv0516c	Rv0516c	up	2.3294568	0.006817216	May be involved in regulating sigma factor	Possible anti-anti-sigma factor	information pathways	Rv0516c-F
BCG_0731	rpsL	Rv0682	rpsL	up	2.1772966	0.002507199	Protein S12 is involved in the translation initiation step.	30S ribosomal protein S12 RpsL	information pathways	Rv0682-R
BCG_1096c	esxJ	Rv1038c	esxJ	up	2.6531754	0.04425926	Function unknown	ESAT-6 like protein EsxJ (ESAT-6 like protein 2)	cell wall and cell processes	Rv1038c-F
BCG_1138c	greA	Rv1080c	greA	up	3.11554	0.003307621	Necessary for efficient RNA polymerase transcription elongation past template-encoded arresting sites. The arresting sites in DNA have the property of trapping a certain fraction of elongating RNA polymerases that pass through, resulting in locked ternary complexes. Cleavage of the nascent trancript by cleavage factors such as GREA or GREB allows the resumption of elongation from the new 3'terminus. GREA releases sequences of 2 to 3 nucleotides	Probable transcription elongation factor GreA (transcript cleavage factor GreA)	information pathways	Rv1080c-F
BCG_1281	sigE	Rv1221	sigE	up	2.252617	0.044271644	The sigma factor is an initiation factor that promotes attachment of the RNA polymerase to specific initiation sites and then is released. Seems to be regulated by sigh (Rv3223c product). Seems to regulate the heat-shock response.	Alternative RNA polymerase sigma factor SigE	information pathways	Rv1221-R

BCG_1823	PE19	Rv1791	PE19	up	2.416305	0.013229125	Function unknown	PE family protein PE19	PE/PPE	Rv1791-R
BCG_1824	esxM	Rv1792	esxM	up	2.7327416	0.034722216	Function unknown	ESAT-6 like protein EsxM	cell wall and cell processes	Rv1792-R
BCG_2139c	hisE	Rv2122c	hisE	up	2.149715	0.005825038	Involved in histidine biosynthesis.	Phosphoribosyl-AMP pyrophosphatase HisE	intermediary metabolism and respiration	Rv2122c-F
BCG_2428	rpsT	Rv2412	rpsT	up	2.1110535	0.006734069	Involved in translation mechanisms. Binds directly to 16S ribosomal RNA.	30S ribosomal protein S20 RpsT	information pathways	Rv2412-R
BCG_2471	BCG_2471	Rv2451	Rv2451	up	2.0198357	0.008232632	Unknown	Hypothetical proline and serine rich protein	conserved hypotheticals	Rv2451-R
BCG_2723	sigB	Rv2710	sigB	up	2.3562243	0.014988984	The sigma factor is an initiation factor that promotes attachment of the RNA polymerase to specific initiation sites and then is released. May control the regulons of stationary phase and general stress resistance. Seems to be regulated by sigh (Rv3223c product) and SIGE (Rv1221 product). Seems to regulate KATG|Rv1908c and the heat-shock response.	RNA polymerase sigma factor SigB	information pathways	Rv2710-R
BCG_3527c	infA	Rv3462c	infA	up	2.2389758	0.00287755	No specific function has so far been attributed to this initiation factor; however, it seems to stimulate more or less all the activities of the other two initiation factors, if-2 and if-3.	Probable translation initiation factor if-1 InfA	information pathways	Rv3462c-F
**BCG ID**	**BCG Name**	**H37Rv ID**	**Rv Name (MB)**	**Regulation**	**FC (abs)**	**p (Corr)**	**Function (MB)**	**Product (MB)**	**Functional Category (MB)**	**Target_ID**
BCG_0061	BCG_0061	Rv0030	Rv0030	down	2.0172732	0.00339416	Unknown	Conserved hypothetical protein	conserved hypotheticals	Rv0030-R
BCG_0147	BCG_0147	Rv0114	gmhB	down	2.525326	0.002507199	Involved in biosynthesis of nucleotide-activated glycero-manno-heptose. Involved in two pathways, D-alpha-D pathway [catalytic activity: D-glycero-alpha-D-manno-heptose 1,7-biphosphate = D-glycero-alpha-D-manno-heptose 1-phosphate] and L-beta-D pathway [catalytic activity: D-glycero-beta-D-manno-heptose 1,7-biphosphate = D-glycero-beta-D-manno-heptose 1-phosphate].	Possible D-alpha,beta-D-heptose-1,7-biphosphate phosphatase GmhB (D-glycero-D-manno-heptose 7-phosphate kinase)	cell wall and cell processes	Rv0114-R

BCG_0207	mce1B	Rv0170	mce1B	down	2.4252424	0.00287755	Unknown, but thought to be involved in host cell invasion.	Mce-family protein Mce1B	virulence, detoxification, adaptation	Rv0170-R
BCG_0233	BCG_0233	Rv0196	Rv0196	down	2.5691757	0.002507199	Possibly involved in transcriptional mechanism.	Possible transcriptional regulatory protein	regulatory proteins	Rv0196-R
BCG_0267c	php	Rv0230c	php	down	2.1670148	0.00287755	Enzymatic activity unknown [catalytic activity: aryl dialkyl phosphate + H2O = dialkyl phosphate + an aryl alcohol].	Probable phosphotriesterase Php (parathion hydrolase) (PTE) (aryldialkylphosphatase) (paraoxonase) (a-esterase) (aryltriphosphatase) (paraoxon hydrolase)	lipid metabolism	Rv0230c-F
BCG_0269	BCG_0269	Rv0232	Rv0232	down	3.1657658	0.012182727	Involved in transcriptional mechanism.	Probable transcriptional regulatory protein (probably TetR/AcrR-family)	regulatory proteins	Rv0232-R
BCG_0270	nrdB	Rv0233	nrdB	down	2.019661	0.003652177	Involved in the DNA replication pathway (first reaction). Provides the precursors necessary for DNA synthesis [catalytic activity: 2'-deoxyribonucleoside diphosphate + oxidized thioredoxin + H2O = ribonucleoside diphosphate + reduced thioredoxin].	Ribonucleoside-diphosphate reductase (beta chain) NrdB (ribonucleotide reductase small chain)	information pathways	Rv0233-R
BCG_0341	BCG_0341	Rv0301	vapC2	down	2.2188537	0.00287755	Unknown	Possible toxin VapC2	virulence, detoxification, adaptation	Rv0301-R
BCG_0342	BCG_0342	Rv0302	Rv0302	down	2.3587484	0.006118542	Involved in transcriptional mechanism.	Probable transcriptional regulatory protein (probably TetR/AcrR-family)	regulatory proteins	Rv0302-R
BCG_0548c	serB1	Rv0505c	serB1	down	2.2508147	0.00287755	Removes a phosphate from phosphoserine [catalytic activity: phosphoserine + H2O = serine + phosphate].	Possible phosphoserine phosphatase SerB1 (PSP) (O-phosphoserine phosphohydrolase) (pspase)	intermediary metabolism and respiration	Rv0505c-F
BCG_0699	BCG_0699	Rv0650	Rv0650	down	2.3578136	0.00287755	Function unknown; probably involved in specific sugar metabolism or regulation.	Possible sugar kinase	intermediary metabolism and respiration	Rv0650-R
BCG_0811c	BCG_0811c	Rv0759c	Rv0759c	down	2.174622	0.005583438	Function unknown	Conserved hypothetical protein	conserved hypotheticals	Rv0759c-F
BCG_0899	lpqS	Rv0847	lpqS	down	2.625553	0.002507199	Unknown	Probable lipoprotein LpqS	cell wall and cell processes	Rv0847-R

BCG_1040c	mscL	Rv0985c	mscL	down	4.0564795	0.002507199	Ion channel that opens in response to stretch forces in the membrane lipid bilayer. May participate in the regulation of osmotic pressure changes within the cell.	Possible large-conductance ion mechanosensitive channel MscL	cell wall and cell processes	Rv0985c-F
BCG_1053	BCG_1053	Rv1190	Rv1190	down	2.3303113	0.004252977	Function unknown	Conserved hypothetical protein	conserved hypotheticals	Rv1190-R
BCG_1068	ispE	Rv1011	ispE	down	2.328591	0.002507199	Thought to be involved in deoxyxylulose-5-phosphate pathway (DXP) of isoprenoid biosynthesis (at the fourth step). Catalyzes the phosphorylation of the position 2 hydroxy group of 4-diphosphocytidyl-2C-methyl-D-erythritol [catalytic activity: ATP + 4-(cytidine 5'-diphospho)-2-C-methyl-D-erythritol = ADP + 2-phospho-4-(cytidine 5'-diphospho)-2-C-methyl-D-erythritol].	Probable 4-diphosphocytidyl-2-C-methyl-D-erythritol kinase IspE (CMK) (4-(cytidine-5'-diphospho)-2-C-methyl-D-erythritol kinase)	intermediary metabolism and respiration	Rv1011-R
BCG_1103	BCG_1103	Rv1045	Rv1045	down	2.0090392	0.004294436	Unknown	Hypothetical protein	conserved hypotheticals	Rv1045-R
BCG_1294	BCG_1294	Rv1234	Rv1234	down	2.7355843	0.002507199	Unknown	Probable transmembrane protein	cell wall and cell processes	Rv1234-R
BCG_1378c	alkA	Rv1317c	alkA	down	2.5348155	0.002990481	Involved in the adaptive response to alkylation damage in DNA caused by alkylating agents. Repairs the SP diastereomer of DNA methylphosphotriester lesions by a direct and irreversible transfer of the methyl group to one of its own cysteine residues. The methylated ALKA protein acts as a positive regulator of its own synthesis, as well as that of other proteins.	Probable bifunctional regulatory protein and DNA repair enzyme AlkA (regulatory protein of adaptative response) (methylphosphotriester -DNA--protein-cysteine S-methyltransferase)	information pathways	Rv1317c-F
BCG_1428	BCG_1428	Rv1366	Rv1366	down	2.8617003	0.002507199	Unknown	Hypothetical protein	conserved hypotheticals	Rv1366-R
BCG_1620c	BCG_1620c	Rv1567c	Rv1567c	down	2.2951014	0.007566907	Unknown	Probable hypothetical membrane protein	cell wall and cell processes	Rv1567c-F
BCG_1780	BCG_1780	Rv1741	vapC34	down	2.4141047	0.002507199	Unknown	Possible toxin VapC34. Contains PIN domain.	virulence, detoxification, adaptation	Rv1741-R
BCG_1809c	BCG_1809c	Rv1776c	Rv1776c	down	2.5397468	0.003397662	Involved in transcriptional mechanism.	Possible transcriptional regulatory protein	regulatory proteins	Rv1776c-F
BCG_1847c	BCG_1847c	Rv1813c	Rv1813c	down	5.443304	0.002507199	Function unknown	Conserved hypothetical protein	conserved hypotheticals	Rv1813c-F
BCG_1849	BCG_1849	Rv1815	Rv1815	down	2.1226277	0.00287755	Function unknown	Conserved protein	conserved hypotheticals	Rv1815-R
BCG_1897	BCG_1897	Rv1861	Rv1861	down	2.5616987	0.003157753	Function unknown	Probable conserved transmembrane protein	cell wall and cell processes	Rv1861-R

BCG_1937	BCG_1937	Rv1898	Rv1898	down	2.4602568	0.005705643	Function unknown	Conserved hypothetical protein	conserved hypotheticals	Rv1898-R
BCG_1942	BCG_1942	Rv1903	Rv1903	down	2.395231	0.00287755	Unknown	Probable conserved membrane protein	cell wall and cell processes	Rv1903-R
BCG_1993c	BCG_1993c	Rv1954c	Rv1954c	down	2.147341	0.00287755	Unknown	Hypothetical protein	conserved hypotheticals	Rv1954c-F
BCG_2004	BCG_2004	Rv1988	erm(37)	down	2.1141493	0.00287755	Thought to cause methylation of 23S rRNA	Probable 23S rRNA methyltransferase Erm(37)	intermediary metabolism and respiration	Rv1988-R
BCG_2059c	BCG_2059c	Rv2040c	Rv2040c	down	2.2476702	0.002507199	Thought to be involved in active transport of sugar across the membrane (import). Responsible for the translocation of the substrate across the membrane.	Probable sugar-transport integral membrane protein ABC transporter	cell wall and cell processes	Rv2040c-F
BCG_2082	BCG_2082	Rv2063	mazE7	down	2.1997433	0.004160331	Function unknown	Antitoxin MazE7	virulence, detoxification, adaptation	Rv2063-R
BCG_2102	BCG_2102	Rv2083	Rv2083	down	2.1493015	0.003274883	Unknown	Conserved hypothetical protein	conserved hypotheticals	Rv2083-R
BCG_2174c	murF	Rv2157c	murF	down	2.0973208	0.00287755	Involved in cell wall formation; peptidoglycan biosynthesis.	Probable UDP-N-acetylmuramoylalanyl-D-glutamyl-2,6-diaminopimelate-D-alanyl-D-alanyl ligase MurF	cell wall and cell processes	Rv2157c-F
BCG_2457c	nadE	Rv2438c	nadE	down	2.0734065	0.003140178	Involved in biosynthesis of NAD. Can use both glutamine or ammonia as a nitrogen source [catalytic activity: ATP + deamido-NAD(+) + L-glutamine + H(2)O = AMP + diphosphate + NAD(+) + L-glutamate].	Glutamine-dependent NAD(+) synthetase NadE (NAD(+) synthase [glutamine-hydrolysing])	intermediary metabolism and respiration	Rv2438c-F
BCG_2574c	BCG_2574c	Rv2551c	Rv2551c	down	2.448835	0.002507199	Function unknown	Conserved hypothetical protein	conserved hypotheticals	Rv2551c-F
BCG_2615c	ruvB	Rv2592c	ruvB	down	2.141356	0.003397662	forms a complex with RUVA. RUVB could possess weak ATPase activity, which will be stimulated by the RUVA protein in the presence of DNA. The RUVA-RUVB complex in the presence of ATP renatures cruciform structure in supercoiled DNA with palindromic sequence, indicating that it may promote strand exchange reactions in homologous recombination. RUVAB is an helicase that mediates the holliday junction migration by localized denaturation and reanneling.	Probable holliday junction DNA helicase RuvB	information pathways	Rv2592c-F

BCG_2666c	BCG_2666c	Rv2639c	Rv2639c	down	2.1540549	0.003274883	Unknown	Probable conserved integral membrane protein	cell wall and cell processes	Rv2639c-F
BCG_2778c	BCG_2778c	Rv2762c	Rv2762c	down	2.7919426	0.003397662	Function unknown	Conserved hypothetical protein	conserved hypotheticals	Rv2762c-F
BCG_2780c	dfrA	Rv2763c	dfrA	down	2.3156297	0.003307621	Essential step for de novo glycine and purine synthesis, DNA precursor synthesis, and for the conversion of dUMP to dTMP [catalytic activity: 5,6,7,8-tetrahydrofolate + NADP(+) = 7,8-dihydrofolate + NADPH].	Dihydrofolate reductase DfrA (DHFR) (tetrahydrofolate dehydrogenase)	intermediary metabolism and respiration	Rv2763c-F
BCG_2804c	ribF	Rv2786c	ribF	down	2.0204735	0.00287755	Involved in FAD biosynthesis [catalytic activity 1: ATP + riboflavin = ADP + FMN] [catalytic activity 2: ATP + FMN = diphosphate + FAD].	Probable bifunctional FAD synthetase/riboflavin biosynthesis protein RibF: riboflavin kinase (flavokinase) + FMN adenylyltransferase (FAD pyrophosphorylase) (FAD synthetase)(FAD diphosphorylase) (flavin adenine dinucleotide synthetase)	intermediary metabolism and respiration	Rv2786c-F
BCG_2928c	rimM	Rv2907c	rimM	down	2.106887	0.00287755	Essential for efficient processing of 16S rRNA. Probably part of the 30S subunit prior to or during the final step in the processing of 16S free 30S ribosomal subunits. It could be some accessory protein needed for efficient assembly of the 30S subunit. RIMM is needed in a step prior to RBFA during the maturation of 16S rRNA. Has affinity for free ribosomal 30S subunits but not for 70S ribosomes.	Probable 16S rRNA processing protein RimM	information pathways	Rv2907c-F
BCG_2945c	BCG_2945c	Rv2923c	Rv2923c	down	2.2120833	0.002507199	Function unknown	Conserved protein	conserved hypotheticals	Rv2923c-F
BCG_3013c	gltS	Rv2992c	gltS	down	2.1587343	0.002507199	Involved in translation mechanisms [catalytic activity: ATP + L-glutamate + tRNA(GLU) = AMP + diphosphate + L-glutamyl-tRNA(GLU)].	Glutamyl-tRNA synthetase GltS (glutamate--tRNA ligase) (glutamyl-tRNA synthase) (GLURS)	information pathways	Rv2992c-F
BCG_3120	BCG_3120	Rv3095	Rv3095	down	2.0427935	0.005279541	Unknown. Could be involved in regulatory mechanism.	Hypothetical transcriptional regulatory protein	regulatory proteins	Rv3095-R

BCG_3166	BCG_3166	Rv3143	Rv3143	down	2.5728054	0.004182312	Unknown, but could be involved in regulatory mechanism	Probable response regulator	regulatory proteins	Rv3143-R
BCG_3189c	BCG_3189c	Rv3165c	Rv3165c	down	2.0405302	0.02486292	Unknown	Unknown protein	conserved hypotheticals	Rv3165c-F
BCG_3327c	lpqC_1	Rv3298c	lpqC	down	2.152159	0.00287755	Function unknown; lipolytic enzyme involved in cellular metabolism.	Possible esterase lipoprotein LpqC	cell wall and cell processes	Rv3298c-F
BCG_3494c	BCG_3494c	Rv3424c	Rv3424c	down	2.2704797	0.011512701	Unknown	Hypothetical protein	conserved hypotheticals	Rv3424c-F
BCG_3567c	fdxD	Rv3503c	fdxD	down	3.0570652	0.00287755	Ferredoxins are iron-sulfur proteins that transfer electrons in a wide variety of metabolic reactions.	Probable ferredoxin FdxD	intermediary metabolism and respiration	Rv3503c-F
BCG_3586	BCG_3586	Rv3522	ltp4	down	2.2292593	0.00287755	Function unknown; probably involved in lipid metabolism.	Possible lipid transfer protein or keto acyl-CoA thiolase Ltp4	lipid metabolism	Rv3522-R
BCG_3611	BCG_3611	Rv3547	ddn	down	2.1071537	0.00287755	Function unknown. Converts bicyclic nitroimidazole drug candidate pa-824 to three metabolites, generating NO.	Deazaflavin-dependent nitroreductase Ddn	intermediary metabolism and respiration	Rv3547-R
BCG_3629	aspB	Rv3565	aspB	down	2.0213947	0.004160331	Thought to be involved in glutamate biosynthesis [catalytic activity: L-aspartate + 2-oxoglutarate = oxaloacetate + L-glutamate].	Possible aspartate aminotransferase AspB (transaminase A) (ASPAT) (glutamic--oxaloacetic transaminase) (glutamic--aspartic transaminase)	intermediary metabolism and respiration	Rv3565-R
BCG_3642	BCG_3642	Rv3577	Rv3577	down	2.2855098	0.00401994	Function unknown	Conserved hypothetical protein	conserved hypotheticals	Rv3577-R
BCG_3698c	BCG_3698c	Rv3640c	Rv3640c	down	2.20298	0.00339416	Required for the transposition of an insertion sequence.	Probable transposase	insertion seqs and phages	Rv3640c-F
BCG_3898	BCG_3898	Rv3835	Rv3835	down	2.2190228	0.003378763	Unknown	Conserved membrane protein	cell wall and cell processes	Rv3835-R
BCG_3903	BCG_3903	Rv3840	Rv3840	down	2.985628	0.002507199	Supposedly involved in transcriptional mechanism.	Possible transcriptional regulatory protein	regulatory proteins	Rv3840-R
BCG_3961c	esxE	Rv3904c	esxE	down	2.4624584	0.002507199	Unknown	Putative ESAT-6 like protein EsxE (hypothetical alanine rich protein) (ESAT-6 like protein 12)	cell wall and cell processes	Rv3904c-F
